# Supplementary material for: Direct imaging of structural changes induced by ionic liquid gating leading to engineered three-dimensional meso-structures
Source: Nat Commun. 2018 Aug 3;9:3055. doi: 10.1038/s41467-018-05330-1 (PMC6076294; doi:10.1038/s41467-018-05330-1)
Supplement: Supplementary file 1 — Supplementary Information [file 41467_2018_5330_MOESM1_ESM.pdf]

Direct imaging of structural changes induced by ionic liquid gating leading to engineered three-dimensional meso-structures

Authors: Cui *et al*

Supplementary Note 1. Growth of epitaxial $SrCoO_{2.5}$ films on (001) $SrTiO_3$

Supplementary Figure 1a shows the $\theta/2\theta$ X-ray diffraction (XRD) pattern of a 40 nm thick $SrCoO_{2.5}$ film deposited on a (001) $SrTiO_3$ (STO) substrate. The sharp peak at 46.48º corresponds to the (002) STO substrate peak while the other main peak at 46.15º is a (008) diffraction peak from the film together with a series of Kiessig thickness fringes. Thus, clear evidence for epitaxial film growth is observed. We find that the *c*-axis lattice parameters for $SrTiO_3$ and $SrCoO_{2.5}$ are equal to 3.905 Å and 15.735 Å, respectively. The rocking curve from the $SrCoO_{2.5}$ (008) diffraction peak in Supplementary Figure 1b shows that the $SrCoO_{2.5}$ thin film is of high crystal quality with a narrow full width at half maximum (FWHM) peak value of 0.045º. The surface morphology of the $SrCoO_{2.5}$ films was studied by atomic force microscopy (AFM, Bruker Dimension Icon). A typical AFM image is shown in Supplementary Figure 1c. The root mean square roughness is ~0.26 nm averaged over an area of 3×3 μm^2.

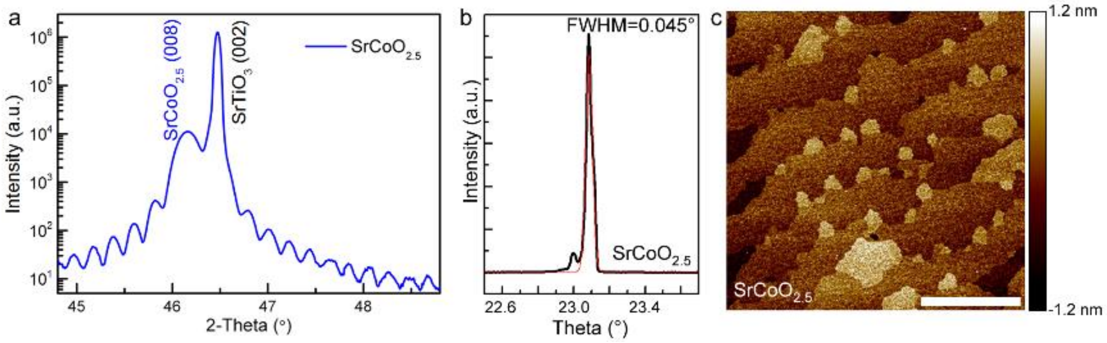

Supplementary Figure 1 | Growth of $SrCoO_{2.5}$ on $SrTiO_3$. a, XRD pattern of a 40 nm thick $SrCoO_{2.5}$ film deposited on a (001) STO substrate. **b**, Corresponding XRD rocking curve from the (008) $SrCoO_{2.5}$ peak. **c**, Typical AFM image of the surface of a $SrCoO_{2.5}$ (40 nm) on (001) STO substrate. The scale bar is 1 μm.

Supplementary Note 2. Transmission Electron Microscopy sample preparation

Thin film lamellae were prepared using conventional cross-section transmission electron microscope (TEM) sample preparation methods. First, two 10×1.5 mm^2 rectangular shaped pieces were cut from a single sample wafer. These pieces are glued together with the film layers facing each other using M-Bond 610 adhesive. Then a cross-sectioned TEM lamella is obtained by thinning the sample, using wire-cutting, mechanical grinding and, finally, argon-ion milling. The final step reduces the thickness of the lamella to no more than 100 nm over an area of several microns. Several cross-section lamellae were fabricated from the same sample and then transferred onto chips for *in-situ* TEM studies. The chips have four independent Pt electrodes, which are used as positive and negative gate voltage electrodes (gold areas in Supplementary Figure 2a, V_G+ and V_G–). After the lamella was mounted on the chip, silver paint (gray areas in Supplementary Figure 2a) was used to connect the negative gate voltage electrodes (V_G–) with the ends of the lamella as shown in the sketch in Supplementary Figure 2a. Finally, a drop of ionic liquid (IL) was placed onto the lamella and precisely moved to a position close to but not on the region to be imaged (red areas in Supplementary Figure 2a) using an AFM tip.

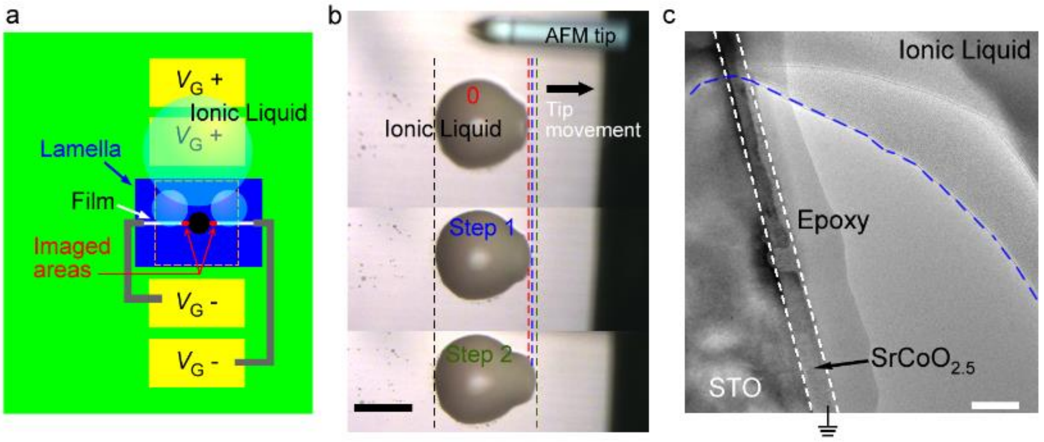

Supplementary Figure 2 | Transmission Electron Microscopy sample preparation. **a**, Sketch of the chip (green), the TEM lamella (blue), and contact electrodes (gold areas for Pt and grey areas for Ag paint). The white line indicates the cross-sectioned film. The black circle corresponds to the hole formed during Ar-ion milling and the two red dots correspond to areas that are imaged in the TEM. The orange dashed frame indicates a hole in the chip for electron beam transmission. **b**, Optical microscopy images in the AFM showing the movement of the IL droplet by the AFM tip in the direction of the black arrow. The droplet moved sideways in the images shown in steps of ~5 μm. The black scar bar is 50 μm. **c**, TEM image of the *in-situ* TEM sample at low magnification. The while scale bar is 100 nm.

The AFM is set to be in the ScanAsyst mode with a sample clearance of 100 μm and scanning probe microscopes (SPM) safety 50 μm. The tip (type SCANASYST-AIR), enters the IL and drags part of it with a minimum step size of ~5 μm, as shown in Supplementary Figure 2b. Both the height and in-plane movement of the AFM tip could be flexibly controlled to achieve the positioning needed. The IL is in contact with the positive gate voltage electrode (V_G+), but not V_G–. The STO

substrate remains highly insulating after fabrication of the lamella, so that any leakage into the substrate during gating is negligible. The chip was then placed onto a special double-tilt sample holder (DENS solutions), where four Pt contact needles are pressed down on the four corresponding Pt electrodes on the chip to both attach the sample fixing and apply a gate voltage. Supplementary Figure 2c shows a TEM image of the *in-situ* TEM sample at low magnification. The edge of the IL is marked by the blue dashed line. During the *in-situ* TEM experiments, a gate voltage (V_G) is applied to the IL. In Supplementary Figure 2c, the surface of the imaged region is free from the IL, so the electron beam can image the crystal structure in this area at high resolution.

Supplementary Note 3. Oxygen concentration changes during *in-situ* gating

The phase transition from $SrCoO_{2.5}$ to $SrCoO_3$ induced by the IL gating is accompanied by an increased oxygen concentration of the thin film, which can be readily seen from energy dispersive x-ray spectroscopy (EDX) measurements that are shown in Supplementary Figure 3. *In-situ* EDX maps were taken within almost the same area for the pristine (Supplementary Figure 3a) and V_G = –3 V gated (12 min) sample (Supplementary Figure 3b). The high angle annular dark field (HAADF) images and the Co and Ti EDX maps clearly show the interface between $SrCoO_x$ (SCO) and STO, which remains stable after gating. The interfaces in the O EDX maps are indicated by red lines. The O EDX map shows a higher oxygen intensity in the sample gated at –3 V, consistent with an increase in oxygen concentration in the thin film after gating. It is noteworthy that the oxygen concentration within the STO

substrate before and after gating does not show any obvious changes, indicating that the injected oxygen in the gated thin film is not derived from the substrate. Taking into consideration the high vacuum environment in the TEM chamber (10^{-8} mbar), the oxygen that is injected into the film during the IL gating most likely comes from O_2 dissolved in the IL.

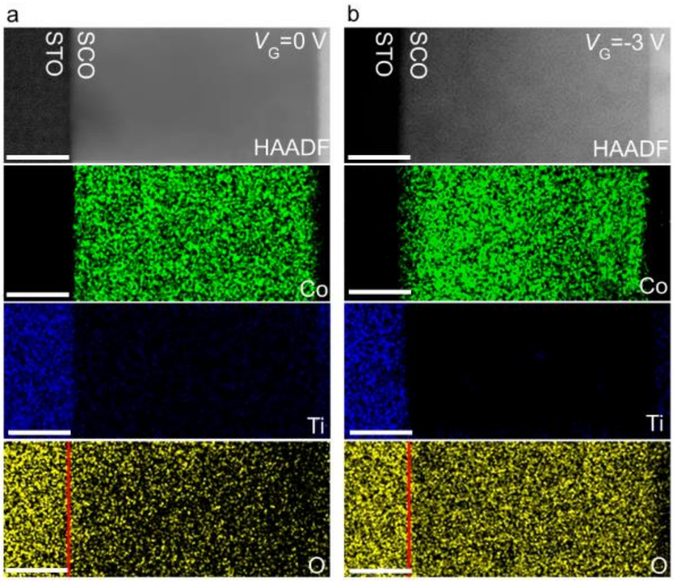

Supplementary Figure 3 | Oxygen concentration variation during *in-situ* gating. *In-situ* EDX of $SrCoO_{2.5}$/STO in its **a**, pristine and **b**, gated states (V_G = –3 V, 12min). The scale bar corresponds to a length of 10 nm.

Supplementary Note 4. Non-volatility of IL gated phase transition

The gating effect is nonvolatile as shown in Supplementary Figure 4. After the phase is fully transformed, the gate voltage is removed. TEM images are shown that were taken just before, and 15 min after the gate voltage was removed. The SCO gated at V_G = –3 V and +2.5 V both show a stable phase after the gate voltage is

removed over an extended period of time, showing the non-volatility of the IL gate induced changes.

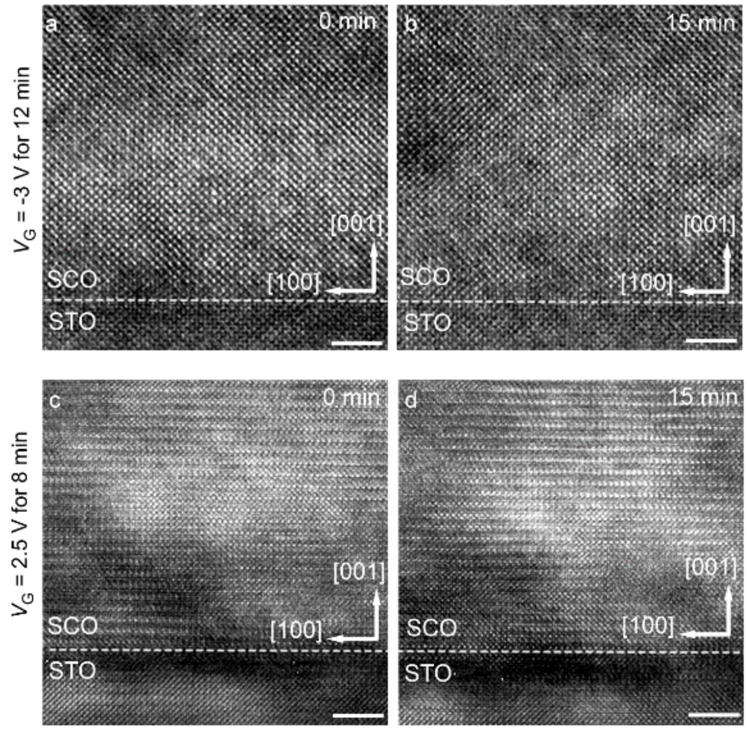

Supplementary Figure 4 | Non-volatility of ionic liquid gate induced phase transition. TEM images for SCO gated at V_G = –3 V: **a**, just before (0 min) and **b**, 15 min after the removal of the gate voltage. TEM images for SCO gated at V_G = +2.5 V: **c**, just before (0 min) and **d**, 15 min after the removal of the gate voltage. The scale bar is 5 nm.

Supplementary Note 5. Crystal structure of large area IL gated thin film samples

Besides the TEM results, the gate controlled phase transition was studied by XRD in large area portions of the same thin film sample used to fabricate the TEM lamellae, as shown in Supplementary Figure 5. The XRD of the pristine $SrCoO_{2.5}$ thin film

(TF-C) clearly exhibits the characteristic doubling of the *c*-axis lattice constant expected for the brownmillerite phase. (002), (006), and (0010) $SrCoO_{2.5}$ diffraction peaks can be clearly identified[1]. For the XRD studies, two thin film samples were gated in comparable conditions to those corresponding to Figure 2o and i in the main text, respectively. Sample TF-A was first gated at V_G = –3, followed by a second gating at V_G = +2.5 V, while sample TF-B was gated at V_G = –3 V. The gating time for each V_G was 30 min. Sample TF-C corresponds to the pristine $SrCoO_{2.5}$ state. For sample TF-B, gating at V_G = –3 V results in the disappearance of the (002), (006) and (0010) $SrCoO_{2.5}$ diffraction peaks, accompanied by shifts of the (004) and (008) diffraction peaks to higher 2θ values. These two peaks then correspond to the (001) and (002) diffractions of the perovskite structure $SrCoO_3$, respectively. Thus, V_G = –3 V induces the phase transition from $SrCoO_{2.5}$ to $SrCoO_3$, consistent with the TEM observations in Figure 2 of the main text. By contrast, sample TF-A, exhibits the characteristic diffraction peaks of $SrCoO_{2.5}$, confirming that the IL gate induced manipulation of the phase transition is reversible. Thus the *ex-situ* XRD gating results on large area thin film samples strongly supports our *in-situ* TEM observations of microscopic areas.

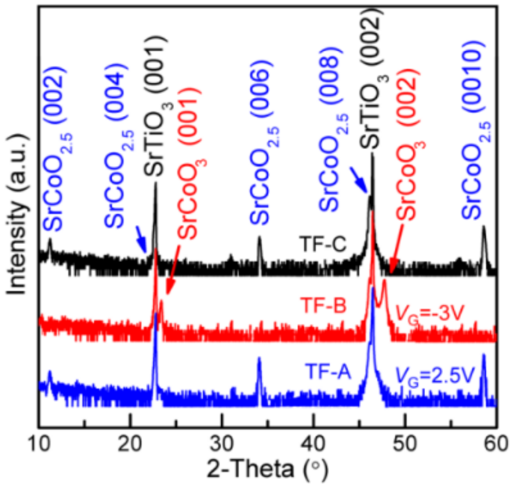

Supplementary Figure 5 | Crystal structure of large area gated thin film samples. *Ex-situ* XRD of $SrCoO_{2.5}$/STO in its pristine and gated states.

Supplementary Note 6. Reversible manipulation of resistance of $SrCoO_x$

Supplementary Figure 6 shows V_G dependent resistance curves for a field effect transistor device made from the same wafer of $SrCoO_{2.5}$ as for the other studies in this paper. The device could be switched reversibly from an insulator to a metal with appropriate gating which is consistent with the known properties of $SrCoO_{2.5}$ and $SrCoO_3$. The resistance of the device could be reversibly switched between two non-volatile states with a resistance change of more than 10^4.

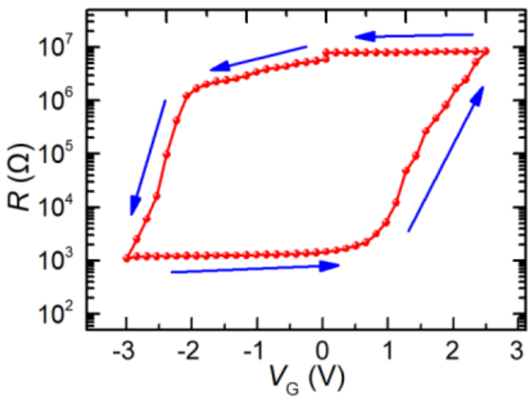

Supplementary Figure 6 | Resistance change based on the phase transition. Gate voltage-dependent resistance of $SrCoO_x$ at 300 K.

Supplementary Note 7. Elimination of electron beam effects on phase transition

To eliminate any possible influence of electron beam irradiation on the IL gate controlled phase transition, control experiments were carried out on both pristine $SrCoO_{2.5}$ (Supplementary Figure 7a) and *in-situ* gated $SrCoO_3$ (V_G = –3 V for 12 mins, Supplementary Figure 7b) lamella as shown in Supplementary Figure 7. The lamellae were exposed to an electron beam (300 kV, ~76.4 nA μm^{-2}, the same as used for the TEM observations) in the TEM for 15 min, which is longer than the longest gating time that we typically used. The crystal structures are clearly unchanged after electron beam irradiation in both $SrCoO_{2.5}$ and $SrCoO_3$ as shown in Supplementary Figure 7. Thus, electron beam irradiation does not play a role in the IL gate driven phase transitions in our *in-situ* TEM experiments[2].

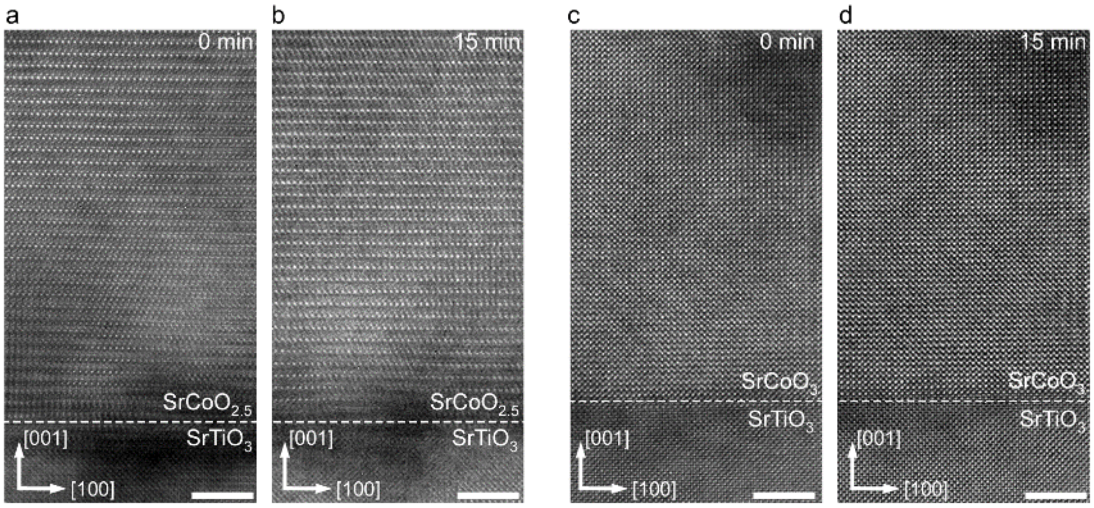

Supplementary Figure 7 | Elimination of electron beam effects on phase transition. TEM images of $SrCoO_{2.5}$ and $SrCoO_3$fordifferent exposure times to the electron beam: **a,** $SrCoO_{2.5}$ 0 min, **b,** $SrCoO_{2.5}$ 15 min, **c,** $SrCoO_3$ 0 min, and **d,** $SrCoO_3$ 15 min. The scale bar is 5 nm.

Supplementary Note 8. TEM results in $SrCoO_{2.5}/(LaAlO_3)_{0.3}(SrAl_{0.5}Ta_{0.5}O_3)_{0.7}$

In addition to our studies of (001) $SrCoO_{2.5}$ (40 nm)/STO samples, *in-situ* TEM observation of IL gating was also carried out for 40 nm thick $SrCoO_{2.5}$ grown on (001) oriented $(LaAlO_3)_{0.3}(SrAl_{0.5}Ta_{0.5}O_3)_{0.7}$ (LSAT) substrate, as shown in Supplementary Figure 8. The in-plane lattice parameters for STO, LSAT, and $SrCoO_{2.5}$ (represented as a pseudo-tetragonal structure) are 3.905 Å, 3.868 Å, and 3.905 Å, respectively[1]. Although $SrCoO_{2.5}$ grown on LSAT undergoes a small compressive strain (~0.9%), as compared to almost zero strain for growth on STO, nevertheless high quality epitaxial and coherent growth of $SrCoO_{2.5}$ films could be achieved (see Supplementary Figure 8a). V_G = –3 V was applied first. With increasing gate time, the area of the brownmillerite SCO phase with the modulated structure seen in the TEM images

becomes steadily smaller, accompanied by ever weaker superlattice reflections in the Fast Fourier transforms (FFTs) shown in the insets to the TEM images in the figures. Thus, this is clear evidence that gating at V_G = –3 V injects oxygen ions into the sample, resulting in the phase transition $SrCoO_{2.5}$→$SrCoO_3$. The phase transition also begins at the surface and extends to the bottom of the thin film, just as we found for similar films grown on STO substrates. The application of V_G for 14 min completes the transition of the brownmillerite SCO to the perovskite SCO in the entire film: this time is also comparable to the SCO films grown on STO. Then a positive gate voltage V_G= +2.5 V was applied to the sample. The SCO phase with a modulated structure is recovered, starting at the surface of the thin film, with a full recovery of the entire film after ~8 min, indicating a $SrCoO_3$→$SrCoO_{2.5}$ phase transition. The different lattice structure of both phases, as seen in these high-resolution TEM images, can be analyzed by the corresponding diffractograms (FFTs). By contrast to $SrCoO_3$, the diffractogram of the $SrCoO_{2.5}$ crystal lattice includes additional reflections, indicated by small yellow circles. In Supplementary Fig. 8a, these additional reflections appear with maximum intensity, but vanish in Supplementary Fig. 8d, indicating a complete transition to the perovskite phase. The reversible manipulation of the phase transition by IL gating in the SCO/LSAT system reaffirms that the massive oxygen reversible transport under IL gating in the SCO system is independent of the substrate and is a property of the SCO film itself.

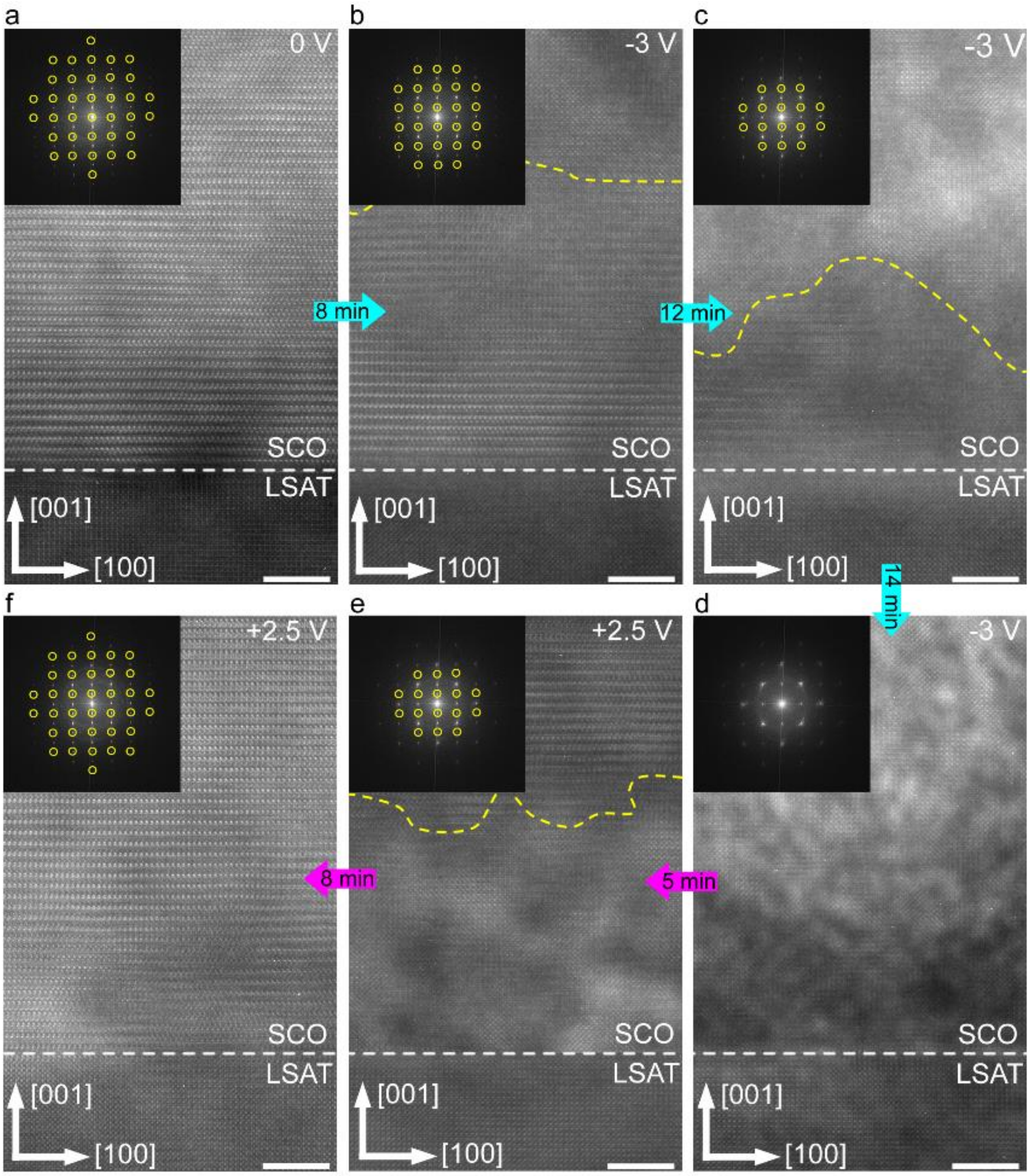

Supplementary Figure 8 | *In-situ* TEM results in $SrCoO_{2.5}$/LSAT. Time-dependent *in-situ* gating TEM images of $SrCoO_{2.5}$ grown on (001) LSAT substrates: **a**, V_G = 0 V, 0 min; **b**, V_G = –3 V, 8 min; **c**, V_G = –3 V, 12 min; **d**, V_G = –3 V, 14 min; **e**, V_G = +2.5 V, 5 min; **f**, V_G = +2.5 V, 8 min. The scale bar is 5 nm. Inserts are the corresponding FFTs.

Supplementary Note 9. Depth-dependent evolution of SCO phases

Depth-dependent line-scan FFTs are performed for the TEM images in main text Figure 2 and shown in Supplementary Figure 9. These results clearly display the evolution of the SCO phases across the thickness of the thin film with time. The line-scan FFT diffractograms in Supplementary Figure 9 correspond to the five TEM images in Figure 2 of the main text, as follows: Supplementary Figure 9a-main text Figure 2c; Supplementary Figure 9b-main text Figure 2e; Supplementary Figure 9c-main text Figure 2g; Supplementary Figure 9d-main text Figure 2k; Supplementary Figure 9e-main text Figure 2m. Each TEM image is divided into 10 sub-areas from the surface to the bottom of the SCO/STO heterostructure with the corresponding FFT diffractograms of each sub-area shown in Supplementary Figure 9. The arrows between the FFT diffractograms indicate the direction from the surface to the bottom of the thin film.

For V_G = –3 V (Supplementary Figure 9a–c), the additional superlattice reflections in the FFT diffractograms become stronger as the line scan moves from the surface to the bottom, showing that the phase transition from $SrCoO_{2.5}$ to $SrCoO_3$ starts from the surface. The amount of brownmillerite SCO can be qualitatively reflected by the ratio of D_{BM}/D_T. The higher the D_{BM}/D_T ratio, the more brownmillerite $SrCoO_{2.5}$ phase is present. The D_{BM} is the intensity of FFT diffractograms caused by brownmillerite phase alone, while the D_T is the intensity of FFT diffractograms caused by both brownmillerite and perovskite phases. The depth-dependent phase transitions in

Supplementary Figure 9a–e are clearly displayed by the evolution of the ratios, $D_{BM}/D_{P}=(D_{BM1}+D_{BM2}+D_{BM3}+D_{BM4})/2\times(D_{T1}+D_{T2})$, which are shown in Supplementary Figure 9f–j. The $D_{BM1–4}$ and $D_{T1–2}$ are the intensity of equivalent FFT spots that arise from the brownmillerite phase and both phases, respectively (see the red and blue arrows in Supplementary Figure 9a). By comparing the depth-dependent D_{BM}/D_{T} ratio evolution in Supplementary Figure 9f–h, it is found that the amount of the $SrCoO_3$ phase increases gradually from the surface to the bottom of the film as the gating time increases. Although the values of D_{BM}/D_{T} for areas 9 in Supplementary Figure 9f–h are slightly lower than those of areas 8, this change in intensity is within the error bar of our measurements. On the other hand, the value of D_{BM}/D_{T} is clearly reduced in area 10, but this is because part of the perovskite $SrTiO_3$ substrate is included in this region of the TEM lamella. Thus, to avoid any misunderstanding, we use open rather than filled symbols for area 10 and use a dashed line to connect these data points with those for area 9 in Supplementary Figure 9f–j.

When V_G = +2.5 V is applied in Supplementary Figure 9d and e, the $SrCoO_{2.5}$ phase begins to extend from the surface to the bottom of the film with increasing gate time. Compared to the FFT diffractograms in Supplementary Figure 9i and j, additional superlattice reflections are found in the region closer to the bottom of the images with a higher D_{BM}/D_{T} ratio as gating time increases. The depth-dependent FFT shows very clearly how the IL gate induced phase transitions start from the surface and extends to the bottom of the thin film. Note that the TEM micrographs do not show an atomically-sharp boundary between the two SCO phases.

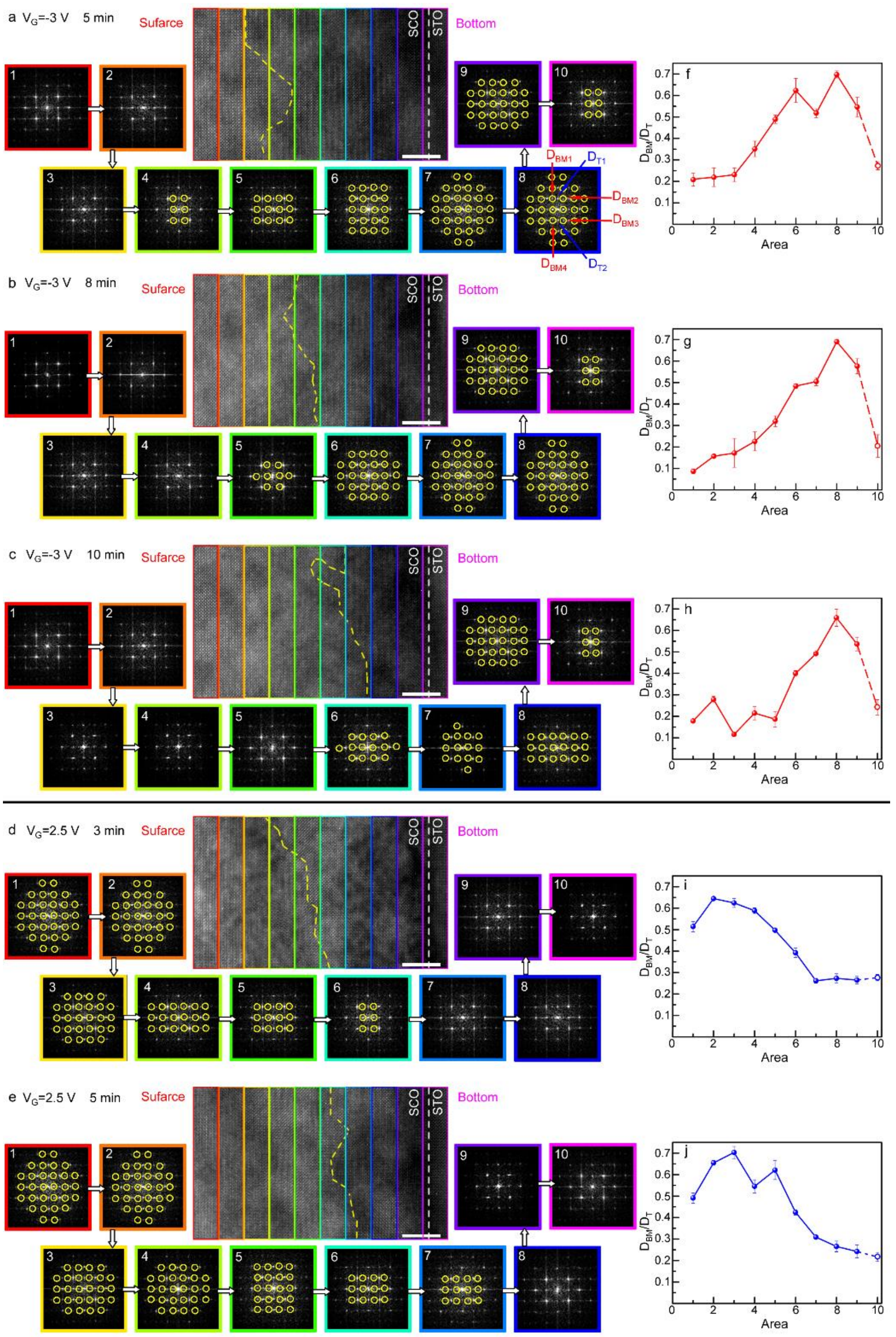

Supplementary Figure 9 | Depth-dependent evolution of $SrCoO_x$ phases. Depth-dependent line-scan FFTs of TEM images from Figure 2 in the main text: **a**, V_G = –3 V, 5 min; **b**, V_G = –3 V, 8 min; **c**, V_G = –3 V, 10 min; **d**, V_G = +2.5 V, 3 min; **e**, V_G = +2.5 V, 5 min. **f–j**, Corresponding ratio of D_{BM}/D_T in **a–e.** The error bar of D_{BM}/D_T is the standard deviation for the following four values: D_{BM1}/D_{T1}, D_{BM2}/D_{T1}, D_{BM3}/D_{T2}, and D_{BM4}/D_{T2}. The scale bar is 5 nm.

Supplementary Note 10. *Ex-situ* X-ray absorption spectra (XAS)

XAS is a powerful tool for investigating the electronic structure and valence state of oxides. *Ex-situ* gated samples were used for these experiments. Here, XAS was performed at 300 K using a total electron yield (TEY) detection mode at the Beamline BL08U1A at the Shanghai Synchrotron Radiation Facility (SSRF). The photon flux is ~8×10^7 s^{-1} on the sample and the beam spot size is 100×100 μm^2. Sample TF-A was gated in the sequence: V_G = –3 V, and then V_G = +2.5 V, while sample TF-B was gated at V_G = –3 V. Each gate voltage was applied for 30 min. Sample TF-C is in the pristine $SrCoO_{2.5}$ state. The O-*K* edge XAS of the pristine and gated SCO are shown in Supplementary Figure 10a. Compared with the pristine sample and TF-A, TF-B shows a strong characteristic peak (highlighted by the yellow shaded region in Supplementary Figure 10a) due to the enhanced Co-O coupling in the high Co oxidizedstate[3,4]. This is evidence for a higher oxygen concentration in TF-B compared with TF-A and TF-C, which is consistent with results from electron energy-loss spectroscopy (EELS). These results support our thesis that oxygen ions are injected into or extracted from the thin films by gating at V_G = –3 and +2.5 V, respectively. Note that the oxygen signals in XAS arise from the SCO thin film (40 nm) rather than the substrate, taking the TEY mode with an attenuation depth of ~6 nm into account[5]. Moreover, as distinct from the samples TF-A and -C, the Co-L_3 peak of sample TF-B shifts to a higher energy (>1.0 eV), suggesting a higher Co valence (Co^{4+}) in this sample (Supplementary Figure 10b).

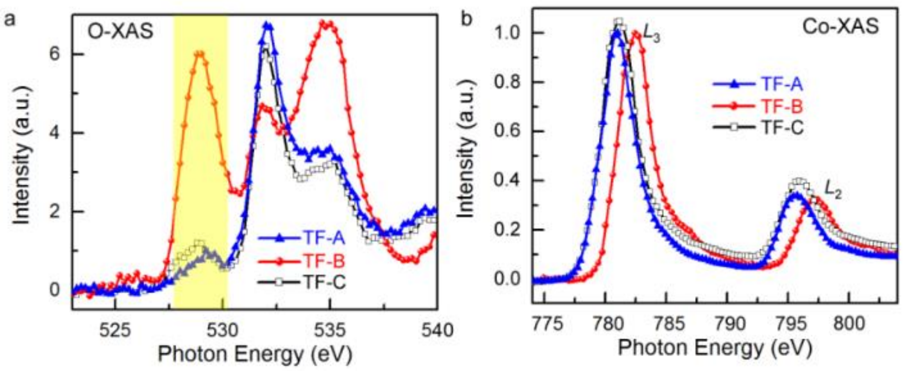

Supplementary Figure 10 | *Ex-situ* X-ray Absorption Spectra. a, O-*K* and **b**, Co-*L*edge XAS of $SrCoO_{2.5}$/STO in pristine and gated states.

Supplementary Note 11. Electron energy-loss magnetic chiral dichroism measurements

Electron energy-loss magnetic chiral dichroism (EMCD) studies were carried out in a TITAN 80-300 STEM at the Ernst Ruska-Centre for Microscopy and Spectroscopy with Electrons Research Centre Jülich[6]. In this EMCD measurement, the experiment was performed in the STEM mode, the beam size used was 1.0–2.0 nm and the semi-convergence angle was ~0.5 mrad[7]. The sample was tilted to the precise two-beam conditions with $(4\bar{4}0)$ systematic reflections excited. Co-$L_{2,3}$ edge EELS data were collected at two chiral positions '+' and '–' in the diffraction plane (inset of Supplementary Figure 11), and the difference spectra that give the dichroic signals (EMCD) were calculated by subtracting the corresponding '+' spectra from the '–' spectra[8]. The EELS and EMCD data shown in Supplementary Figure 11 were obtained at 95 K with the objective lens on and a magnetic field of 2 T applied along the electron beam direction. Comparison of the intensity changes of both Co-L_3 and L_2 edges at the '+' and '–' positions (the dichroism spectra shown in Supplementary

Figure 11) mainly describes the difference between the spin populations. A chiral dichroism signal is observed for the Co-$L_{2,3}$ edges in sample TF-B, but not in sample TF-A, consistent with the ferromagnetic nature of bulk $SrCoO_3$ and the antiferromagnetic nature of bulk $SrCoO_{2.5}$ (Ref. 1).

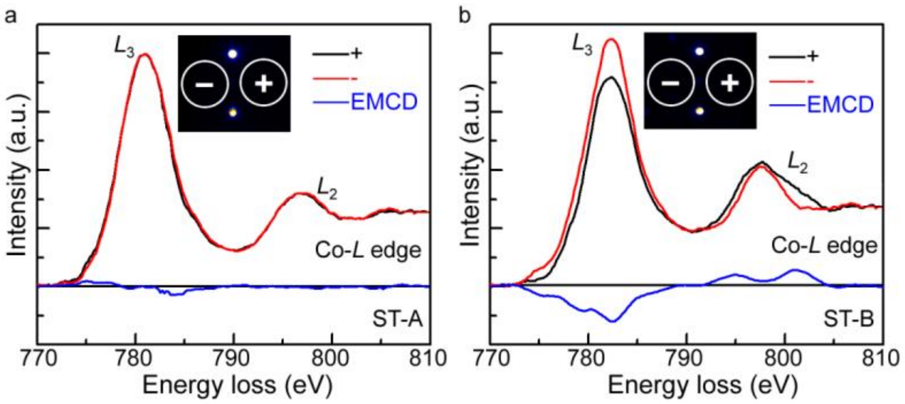

Supplementary Figure 11 | Electron energy-loss magnetic chiral dichroism. EELS measured at the two chiral positions"+" and "–" under the $(4\bar{4}0)$ two beam conditions at diffraction plane, are shown as black and red curves. The difference between the curves, shown as the blue curve, is the EMCD signal. EELS and EMCD are shown for **a**, sample ST-A and **b**, sample ST-B. The inset shows the symmetrically excited diffraction spots (000) and $(4\bar{4}0)$ in the experiment.

Supplementary Note 12. Magnetic properties of the gated thin film samples

The magnetic properties of 3 large area thin film samples are shown in Supplementary Figure 12a and b. The samples TF-A and TF-C are both non-magnetic. On the other hand, TF-B which was gated at V_G = –3 V shows a substantial saturation magnetization (69.8 emu cm^{-3}) with a Curie temperature of ~225 K, consistent with

the known magnetic ferromagnetic character of $SrCoO_3$. The transport and magnetization results support the crystal and electronic structure changes obtained by (S)TEM.

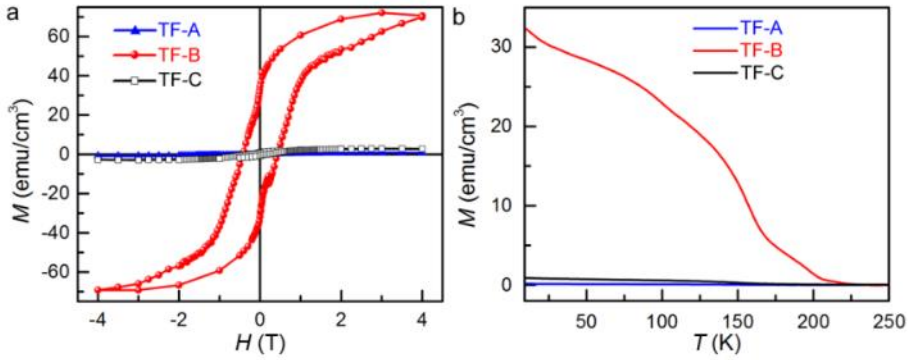

Supplementary Figure 12 | Magnetic properties of the gated thin film samples. a, Magnetization (*M*) versus magnetic field *H* (*T* = 10 K) and **b**, *M* versus *T* (*H* = 5mT) for pristine and IL gated samples.

Supplementary Note 13. Interruption of IL gating effect by resist

The electron beam resist (positive resist ZEP520A ~400 nm thick) were used to block the IL gating effect vertically. For example, the pristine VO_2 is in the insulating state at room temperature, reflected by a low current intensity (blue color) in conductive AFM (CAFM) image (Supplementary Figure 13a). Then the resist was spin-coated on this sample. In this case, the resist fully covered the surface of the sample without any orifices and V_G of +2.5 V was applied on it through IL for 2 hours. Subsequently, both IL and resist were clearly removed by ultrasonic cleaning in acetone and isopropanol. The CAFM image of the sample after this treatment shows homogeneous insulating character in Supplementary Figure 13b, which is similar to the case of the pristine state. On the contrary, for the same sample which was gated by V_G = +2.5 V for 2 hours without any resist, one can clearly see the uniform IL gating induced conductive character (high current, green color) as shown in Supplementary Figure 13c. For $La_{0.45}Sr_{0.55}MnO_3$ and $SrCoO_{2.5}$, V_G = +2.5 V and –3 V could drive them from the pristine high-resistance states (Supplementary Figure 13d and g) to the low-resistance states (Supplementary Figure 13f and i), respectively. However, even after IL gating, the resist covered samples in Supplementary Figure 13e and h do not show any distinct conductive characters compared with the pristine ones. All the results in these three materials suggest that the resist could effectively block the IL gating effect right above.

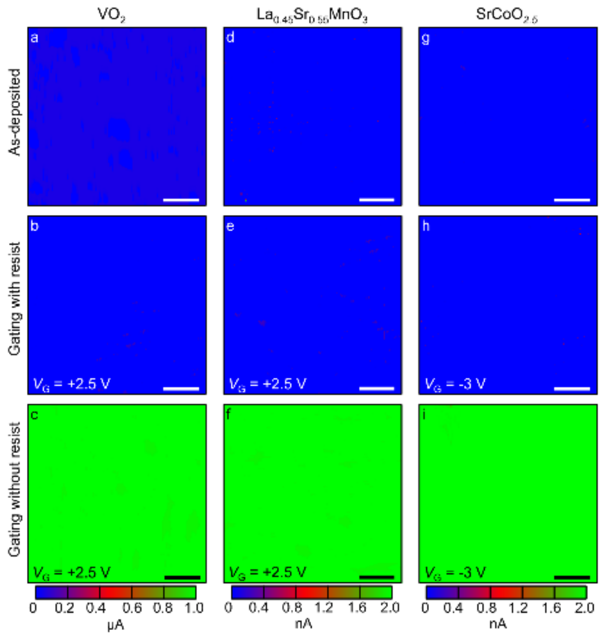

Supplementary Figure 13 | Interruption of IL gating effect by resist. Typical CAFM images for VO_2: **a**, pristine states, **b**, after 2 h gating under V_G = +2.5 V with fully covered resist, and **c**, after 2 h gating under V_G = +2.5 V without resist; Typical CAFM images for $La_{0.45}Sr_{0.55}MnO_3$: **d**, pristine states, **e**, after 2 h gating under V_G = +2.5 V with fully covered resist, and **f**, after 2 h gating under V_G = +2.5 V without resist; Typical CAFM images for $SrCoO_{2.5}$: **g,** pristine states, **h,** after 2 h gating under V_G = –3 V with fully covered resist, and **i,** after 2 h gating under V_G = –3 V without resist. The scale bar is 1 μm.

Supplementary Note 14. Patterned ionic liquid gating and CAFM measurements

Resist orifices were constructed on the samples by exposing a positive resist ZEP520A (~400 nm thick), using electron beam lithography (Raith PIONEER Two). The AFM images shows the morphology of a typical $SrCoO_{2.5}$ sample with resist orifices, which exhibits sharp arrays of orifices 1 μm in diameter and spaced 2 μm

apart (Supplementary Figure 14a). Nine 200×200 arrays were made on a 5×5 mm^2 thin film sample, which was then separated by wire cutting and used for IL gating individually. Samples with resist orifices were IL gated, as shown in Supplementary Figure 14b. The edges of the samples were surrounded by insulating paraffin, so that the IL was not in direct contact with the conducting substrate. Hence the leakage current from IL to the conducting substrate was avoided in the gating process and the effective electric double layer only formed at the interface between the IL and the thin film. At one edge far away from the Au electrode, the thin film and conductive substrate was exposed by scratching the surface resist using a knife. The IL covered almost the whole surface of the sample and Au gate electrode, except for the scratched edge of the sample. In a probe station (Lake Shore), two probes respectively contacted the surface of the scratched region on the sample and the Au electrode to apply gate voltages during IL gating. After the gating process, the IL, resist, and paraffin could be easily removed by ultrasonic cleaning in acetone and isopropanol. Samples were then affixed to a metal holder by conductive silver paint on the bottom of the samples. In these measurements, a 1 MΩ resistor was connected in series with the VO_2 sample, while a 500 MΩ resistor was connected in series with the $La_{0.45}Sr_{0.55}MnO_3$ and SCO samples, as shown in Supplementary Figure 14c. A low noise amplifier (typical noise 1.5 pA) was used to carry out the measurements at a constant voltage of 1 V. The CAFM function in our Cypher (Asylum Research) instrument was used to measure the current flowing across the sample (perpendicular to the surface). A silicon tip with Ti/Ir coating (Asyelec-01) was used and the radius of the tip was 28±10 nm.

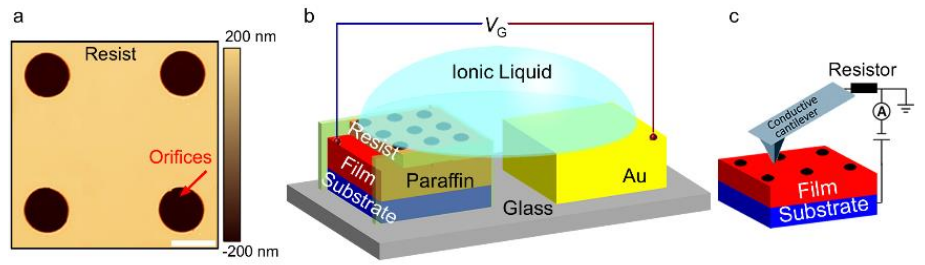

Supplementary Figure 14| Sketch of gating experimental set-up for CAFM measurements. a, Topography of $SrCoO_{2.5}$/NSTO with resist orifices. The scale bar is 1 μm. **b**, Sketch of IL gating before CAFM measurement. **c**, Schematic of CAFM measurement.

Supplementary Note 15. Ionic liquid gating effect on VO_2 and $La_{0.45}Sr_{0.55}MnO_3$

The IL gating effect on a 20 nm (001) VO_2 film deposited on TiO_2 substrate and a 20 nm (001) $La_{0.45}Sr_{0.55}MnO_3$ film deposited on an STO substrate were investigated as shown in Supplementary Figure 15. The temperature dependence of the resistance of VO_2 and $La_{0.45}Sr_{0.55}MnO_3$ was measured in a transistor device which was also used for $SrCoO_{2.5}$. No resist patterns were used in these measurements, so that IL gating modulated the resistance of the entire channel in the transistor device. The pristine VO_2 shows a metal-insulator transition (MIT) with a transition temperature above room temperature. The MIT of VO_2 is suppressed at V_G = + 2.5 V, accompanied by a low resistance state over the range of temperature here between 100 and 400 K. On the other hand, the resistance of $La_{0.45}Sr_{0.55}MnO_3$ is dramatically reduced when a V_G of + 2.5 V is applied through IL. Around three orders of magnitude resistance reduction after IL gating is observed in both materials at room temperature, which is attributed to extraction of oxygen induced by thee IL gating[9,10].

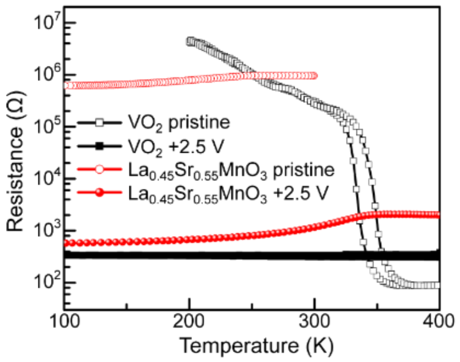

Supplementary Figure 15 | Ionic liquid gating effect on VO_2 and $La_{0.45}Sr_{0.55}MnO_3$. Temperature dependent resistance of VO_2 (cooling and warming) and $La_{0.45}Sr_{0.55}MnO_3$ (cooling) in pristine state and gated by V_G = +2.5 V.

Supplementary Note 16. Conductive AFM results

To demonstrate the possibility of the creation of complex meso-structures by local IL gating, we have created patterns of orifices in resist layers that were spin-coated on to several distinct oxide films that show different degrees of anisotropic oxygen ion transport. Here we present data on IL gated (001) oriented VO_2, $La_{0.45}Sr_{0.55}MnO_3$, and $SrCoO_{2.5}$ films covered by resist layers that were patterned using electron beam lithography to have orifices 1 μm in diameter spaced 2 μm apart. The resist is covered by the IL but the oxide films are only subjected to the IL within the orifices. Thus, on gating an intense electric field can only be formed within these limited regions at the oxide surface. After the application of V_G = +2.5 V on VO_2 and $La_{0.45}Sr_{0.55}MnO_3$ while V_G = –3 V on $SrCoO_{2.5}$ for 5 min and 2 h, the IL and resist pattern were removed to perform the following CAFM measurements. The SCO samples used here are from the same wafer as those in Figure 5 of the main text.

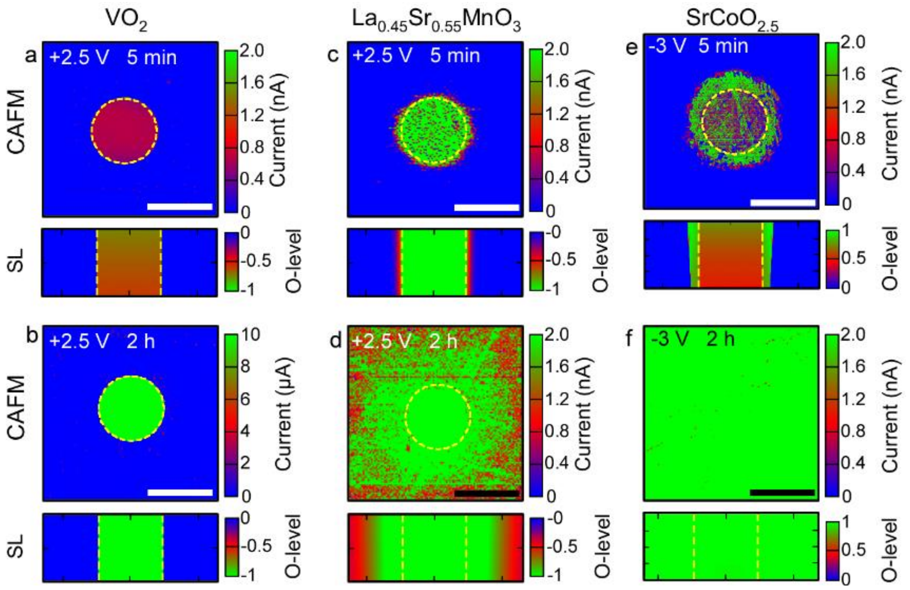

Supplementary Figure 16 | Conductive AFM results for various gating times. Typical CAFM images and simulation results (SL) of oxidation level (O-level) for VO_2, $La_{0.45}Sr_{0.55}MnO_3$, and $SrCoO_{2.5}$ after IL gating through resist orifices patterns. V_G = +2.5 V for 5 min/2 h on **a/b,** VO_2 and **c/d**, $La_{0.45}Sr_{0.55}MnO_3$ and V_G = –3 V for 5 min/2 h on **e/f**, $SrCoO_{2.5}$ film. The scale bar is 1 μm.

The pristine films of (001) VO_2 display a metal-insulator transition above room temperature (Supplementary Figure 15), so the films are poorly conducting at ambient temperature. After IL gating, we find clear evidence that only within the orifice is there a change in the film conductivity with a substantial increase in conductivity, while the regions covered by resist are not affected at all by the IL gating. As the gating time increases from 5 min to 2 h, the metallic state is gradually enhanced but strictly limited to the resist orifices (marked by the yellow dashed circle) due to the highly anisotropic oxygen transport that only takes place along [001][11] (Supplementary Figure 16a and b). By contrast, oxygen transport in $La_{0.45}Sr_{0.55}MnO_3$

is known to take place along all three principal crystallographic directions[12,13]. In this case we find that the initially high-resistance film becomes conducting not only within but well beyond the circumference of the orifices with increasing gate time (Supplementary Figure 16c and d). Finally, for the films of $SrCoO_{2.5}$ a more complex conductivity pattern is found after gating with increases in conductivity well beyond the orifice, as for $La_{0.45}Sr_{0.55}MnO_3$, but with a ring of maximum conductivity at the edge of the orifice after 5 min gating (Supplementary Figure 16e). As the gating time increases to 2 h, the whole area of the thin film becomes conductive as shown in Supplementary Figure 16f.

Supplementary Note 17. Microstructure of SCO after IL gating with resist pattern

The microstructure of the $SrCoO_{2.5}$ thin film after IL gating through resist orifices pattern was investigated by *ex-situ* (S)TEM (scanning TEM) as shown in Supplementary Figure 17. The film with resist orifices was gated under V_G = –3 V for 15 min and then the IL was removed by ultrasonic cleaning in deionized water. A Pt layer of ~20 nm was deposited on the sample with a subsequent lift-off process. Then, a small part of sample was cut along the orifice diameter direction and thinned both-sides by a standard focused ion beam technique to make a suitable (S)TEM lamella cross-section. The previous resist orifices were covered by Pt, to serve as marks in the TEM images as shown in Supplementary Figure 17a. The STEM-HAADF (high angle annular dark field) images of three different areas: (1)

~400 nm, (2) ~200 nm away from the edge of previous resist orifice, and (3) in the region of the resist orifice are shown in Supplementary Figures 17b–d, respectively. The brownmillerite phase, a brownmillerite/perovskite mixture phase, and the perovskite phase are found in areas (1), (2), and (3), respectively. The interface between the brownmillerite/perovskite phases in Supplementary Figure 17c is approximately indicated by the yellow dashed line. Area (1) is outside the insulating region in Figure 5c of main text, while area (2) corresponds to the highest conducting region and area (3) corresponds to the medium conducting region.

We extracted the average *c*-lattice parameters of brownmillerite and perovskite phases in these three areas as summarized in Supplementary Figure 17e. The *c*-lattice parameters of areas (1) and (3) are obtained from the whole image. While the *c*-lattice parameters of brownmillerite and perovskite phases in area (2) are respectively obtained from the regions (2)-1 and (2)-2 (marked by the red dashed frames). The *c*-lattice values of brownmillerite phases are multiplied by 1/4. The 1/4 brownmillerite *c*-lattice of area (1) is 3.925 Å, which is close to the value of 3.934 Å in the initial $SrCoO_{2.5}$ thin film (see XRD results in Supplementary Figure 1). While the *c*-lattice of area (2)-1 near the phase boundary is only 3.874 Å, which might be caused by a clamping effect from the adjacent perovskite phase and a slightly enhanced O-level[14]. It is noteworthy that, for the perovskite phases, the *c*-lattice of area (2)-2 is only 3.815 Å, which is even lower than that of area (3) (c = 3.848 Å). The lower *c*-lattice in area (2)-2 suggests a higher O-level in this area compared with that in area (3)[14], in line with the highest conductivity found from CAFM.

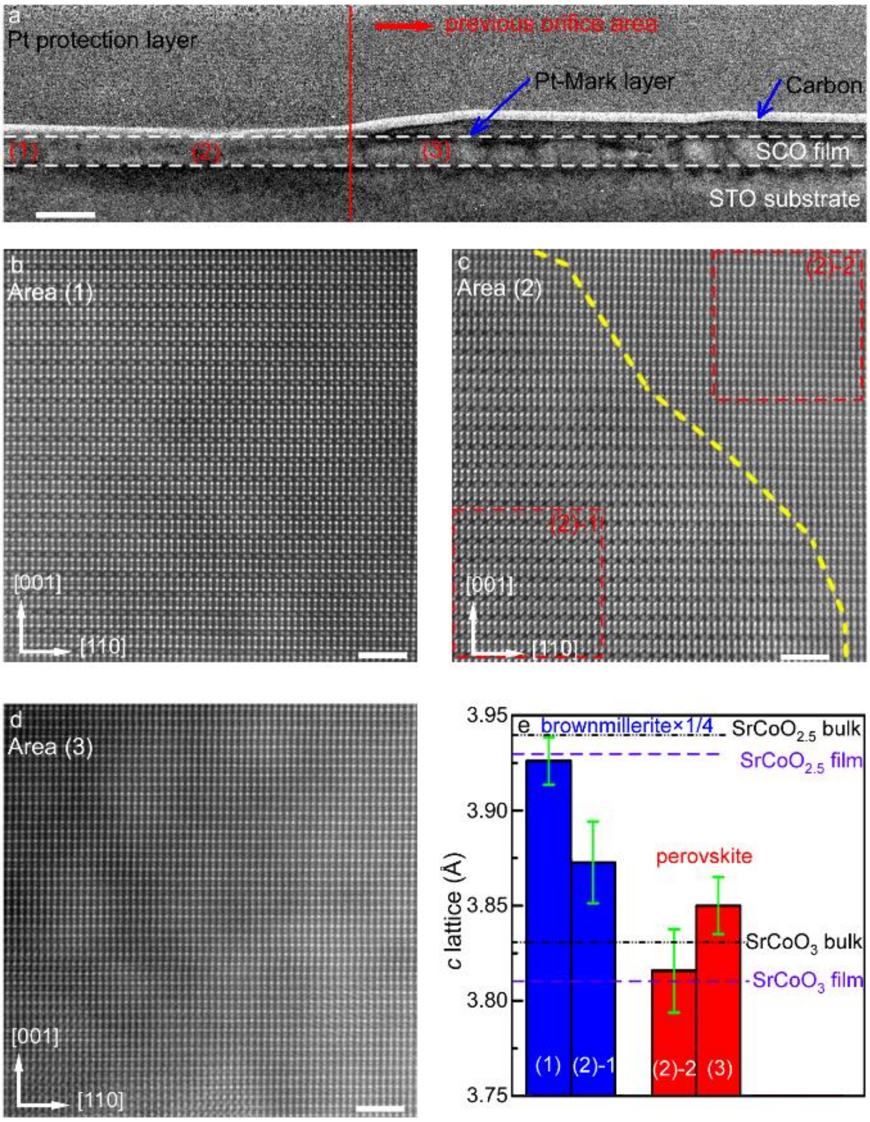

Supplementary Figure 17 | Microstructure of $SrCoO_x$ after gating through a resist pattern. a, The TEM overview image of the $SrCoO_{2.5}$ thin film after IL gating through resist orifices. The scale bar in **a** is 100 nm. Enlarged STEM-HAADF images of three different areas in **a**: **b**, Area (1), **c**, Area (2), and **d**, Area (3). The brownmillerite/perovskite phase boundary in **c** is roughly marked by the yellow dashed line. The red dashed frames in **c** indicate the region in which the c lattice parameter was estimated. The scale bars in **b–d** are 2 nm. **e**, *c*-lattice parameters for of brownmillerite (multiplied by 1/4) and perovskite phases in three different areas. The bulk values are from Ref. 1 and film values are from our XRD measurements.

Supplementary Note 18. Simulation of oxygen level in gated thin films

A two-dimensional model based on diffusion of oxygen derived from gradients in the oxygen concentration was developed to account for the IL gate induced oxygen distributions. Simulations were carried out using a finite element model. The simulation was carried out in a 2D plane in which the *x* and *z* axes are parallel and perpendicular to the surface of the thin film, respectively, as shown in Supplementary Figure 18a. An area of size L_x by L_z was modelled with boundary conditions as discussed below. The finite elements of size $\Delta l \times \Delta l$ were chosen to be 1 nm × 1 nm. The orifice through which the oxygen ions are injected corresponds in our 2D model to a line at $z = L_z$ centered at $x = L_x/2$ with a length defined by L_{inj} as follows: $L_{\mathrm{inj}}/2<(x–L_x/2)<+L_{\mathrm{inj}}/2$ and $z = L_z$. L_{inj} corresponds to the diameter of the resist orifice in our experiments.

$O^{2–}$ are injected into $SrCoO_{2.5}$ at negative V_G while V_O are injected into VO_2 and $La_{0.45}Sr_{0.55}MnO_3$ at positive V_G. The maximum and minimum oxygen levels of any given element in the simulation are set to be those found experimentally. It has been reported that IL gating leads to a reduction in oxygen content in VO_2, and (La,Sr)MnO_3, by respectively, ~3% (Ref. 9), and ~5% (Ref. 10). Here we report a 17% increase in oxygen content for $SrCoO_{2.5}$ when gated to $SrCoO_3$, so that the maximum variation in the total oxygen content corresponds in our simulation to –0.06 from a maximum oxygen content of O_2 for VO_2, –0.15 from a maximum oxygen content of O_3 for $La_{0.45}Sr_{0.55}MnO_3$, and +0.50 from a minimum oxygen content of $O_{2.5}$ for $SrCoO_{2.5}$. In the simulation these bounds are thus used to constrain the possible

variations in oxygen level (O-level). For convenience we normalize the O-level by these experimental bounds so that the O-level $C(x,z,t)$ varies between 0 and 1 for $SrCoO_{2.5}$ and 0 and –1 for VO_2 and (La,Sr)MnO_3. Initially $C(x,z,t=0)$ is set to be zero. Changes in C are calculated for each element at time steps of Δt, that corresponds to one second in our simulations, for a total of 10^4 time steps. Starting at $t = 0$, O^{2-} or V_O are injected for the elements corresponding to $-L_{inj}/2<(x-L_x/2)<+L_{inj}/2$ and $z = L_z$ so that for these elements:

$$C(t + \Delta t) = C(t) + R_{\text{inj}} \cdot \Delta t \quad (1)$$

where R_{inj} is the injection rate of O^{2-} or V_O per element in the orifice. R_{inj} is positive and negative for the injection of O^{2-} and V_O, respectively. Then the O-level gradient ΔC is calculated as follows:

in the $\pm x$ directions: $\Delta C_{\pm x} = \frac{C(x\pm\Delta l,z)-C(x,z)}{\Delta l}$ (2)

in the $\pm z$ directions: $\Delta C_{\pm z} = \frac{C(x,z\pm\Delta l)-C(x,z)}{\Delta l}$ (3)

ΔC at the boundaries and corners of the model are set to be 0. As the injection of O^{2-} into $SrCoO_{2.5}$ induces a change in crystal structure from brownmillerite to perovskite, we introduce an interface between these two phases (a phase-boundary). The critical value of C in our model where this phase transition takes place is set to be $C = 0.5$ corresponding to x=2.75 for $SrCoO_x$[15]. We define the phase boundary to be at one or other of the elements between which $C = 0.5$, depending on the $\pm x$ or $\pm z$ directions, as shown in Supplementary Figure 18c. We make an important assumption that for oxygen to cross this phase boundary, the difference in the O-levels between the perovskite and brownmillerite elements on either side of this boundary should

exceed a threshold value that we define to be δ_t. When the difference of the O-levels across the phase boundary is less than δ_t, we set the corresponding $\Delta C_{\pm x \text{ or } \pm z}$ to be 0.

In our model we use distinct coefficients for oxygen diffusion along the x and z directions (D_x and D_z) using values taken from the literature[12,13,16–18]. At the phase boundary there is likely to be band bending[19,20] which will lead to a local excess or deficiency in oxygen concentration. This, we assume, will lead to an asymmetry in oxygen transport across the phase boundary (Supplementary Figure 18c). The diffusion coefficients at the phase boundary are thus modified by ΔD, as follows:

$$D_{\pm x \text{ or } \pm z}|_{\text{interface}} = D_{x \text{ or } z} \pm \Delta D \cdot u \quad (4)$$

where u takes values of +1 or –1 depending on the direction of the phase boundary. Then, the diffusion of oxygen at each time step is calculated using Fick's second law, as follows:

$$\frac{C(t+\Delta t)-C(t)}{\Delta t} = \frac{1}{\Delta l}(D_{+x}\Delta C_{+x} + D_{-x}\Delta C_{-x} + D_{+z}\Delta C_{+z} + D_{-z}\Delta C_{-z}) \quad (5)$$

Typical simulation results for $SrCoO_{2.5}$ at various times are shown in Supplementary Figure 18. All the parameters used in the simulation are summarized in Supplementary Table 1.

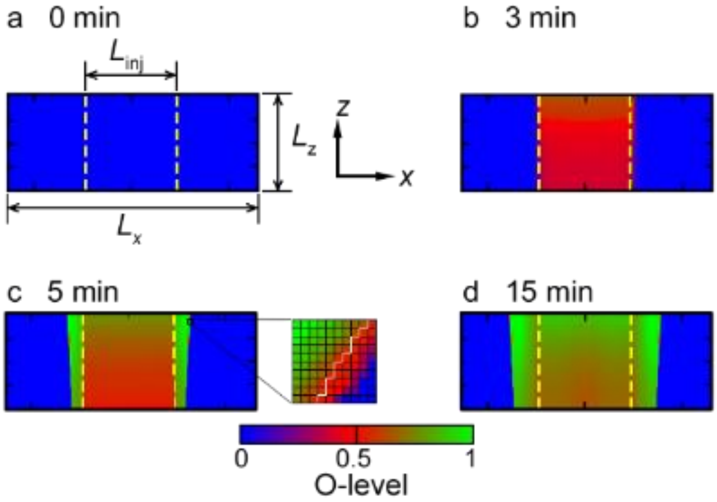

Supplementary Figure 18 | Simulation of oxygen level in gated thin films. Results of the simulation for $SrCoO_{2.5}$ at times **a**, 0 min, **b**, 3 min, **c**, 5 min, and **d**, 15 min. The yellow dashed lines indicate the area of the film below the orifice. The sketch at the right side of **c** shows the phase boundary area in detail. The white solid line indicates the phase boundary between the perovskite and brownmillerite phases.

Supplementary Table 1 Parameters for oxygen level simulation

Materials	VO_2	$La_{0.45}Sr_{0.55}MnO_3$	$SrCoO_{2.5}$
L_x (m)	3×10^{-6}	3×10^{-6}	3×10^{-6}
L_z (m)	2×10^{-8}	2×10^{-8}	4×10^{-8}
$L_{orifice}$ (m)	1×10^{-6}	1×10^{-6}	1×10^{-6}
$R_{inj.}$ (s^{-1})	–0.01	–0.01	0.01
D_x (m^2s^{-1})	0	5×10^{-18}	Surface: 4×10^{-15}<br>Bulk: 4×10^{-17}
D_z (m^2s^{-1})	2×10^{-18}	5×10^{-18}	4×10^{-17}
δ_t	0	0	0.2
ΔD (m^2s^{-1})	0	0	2×10^{-15}

The O-level simulation results in Supplementary Figure 16 are well in line with the corresponding CAFM images. To keep the same color contrasts in CAFM and simulation results, the O-level color contrasts used for VO_2 and $La_{0.45}Sr_{0.55}MnO_3$ are opposite to that of $SrCoO_{2.5}$. The simulations qualitatively account for the distinct conductivity patterns observed experimentally in the three different oxide films. The VO_2 with a [001] oxygen transport channel along z shows a reduction in O-level only below the V_O input line even as the simulation time steps are increased. On the contrary, $La_{0.45}Sr_{0.55}MnO_3$, with both in-plane and out of plane oxygen transport, shows a reduction in O-level beyond the area below the input line, which becomes

more pronounced over time. In SCO, O^{2-} is locally accumulated at the interface between the brownmillerite (low O-level) and perovskite (high O-level) phases, resulting in a region with higher O-level than that of the O^{2-} injection orifice. Although the brownmillerite/perovskite interface also exists along the lateral direction (i.e. along x) in the orifice area, no oxygen accumulation region is found there, because of the smaller oxygen diffusion rate along z. As the simulation time-steps increase, a uniform enhancement of the O-level is found in the whole area of the SCO sample. The simulation results demonstrate that the IL gate induced conductivity distributions of these three materials are strongly related to the O-level variations induced by IL gating. In the simulations for SCO, we find that the phase boundary moves ~40.4 times faster laterally than vertically, which is comparable to our experimental finding of ~34.5 times.

Supplementary Note 19. Sensitivity of simulation results to model parameters

Typical O-level profiles along the x direction of $SrCoO_{2.5}$ at different depths below the surface and their sum are shown in Supplementary Figure 19a at a simulation time of 15 min. One can clearly see regions with higher O-level than that within the O^{2-} injection orifice, which we describe as a high O-level region (HOR, shaded area in Supplementary Figure 19a). As the O-level is symmetric with respect to $x = L_x/2$, we only consider the HOR region on the left. We define the excess O-level within the HOR region as the average O-level difference between the O^{2-} injection orifice and that within the HOR region: we term this the HOR intensity. We term the

length of the HOR region along the *x* direction to be the HOR width (see Supplementary Figure 19a). With increasing simulation time steps, both the intensity and the width of the HOR first increase and then decrease (see Supplementary Figures 19b, c, f, and g). The maximum intensities and widths are summarized in Supplementary Figures 19d, and h. The simulation time step when the HOR first appears and its lifetime (i.e. the time step where it disappears) are summarized in Supplementary Figure 19e, and i.

By changing the values of δ_t and R_{inj} in the simulation, the maximum intensity, width, the time when the HOR first appears (App. time) and the lifetime can be varied, as illustrated in Supplementary Figure 19. In Supplementary Figures 19b, and c all parameters except for δ_t are the same as those in Supplementary Table 1 for $SrCoO_{2.5}$. As δ_t is varied from 0.02 to 0.5, the HOR maximum intensity and width (Supplementary Figure 19d) significantly increase from 0.02 to 0.42 and from 31 nm to 1412 nm, respectively. Meanwhile, as δ_t is increased, the App. time increases from 4 to 7 min and the lifetime is dramatically increased from 7 to 38 min (see Supplementary Figure 19e). We now consider the influence of R_{inj} on the behavior of HOR. Variations in R_{inj} can be realized by varying the gate voltage in the experiments. As R_{inj} is increased from 10^{-3} s^{-1} to 10 s^{-1} (all the other parameters are the same as those in Supplementary Table 1 for SCO), the maximum intensity and width of the HOR is sharply reduced from 0.44 to 0.016, and from 1462 nm to 40 nm, respectively (see Supplementary Figure 19h). The App. time and lifetime of the HOR are reduced from 88 min to 0, and from 192 min to 0, respectively (Supplementary Figure 19i).

These results suggest that the HOR can be effectively controlled by changing the oxygen injection rate in the IL gating process by varying the magnitude of V_G.

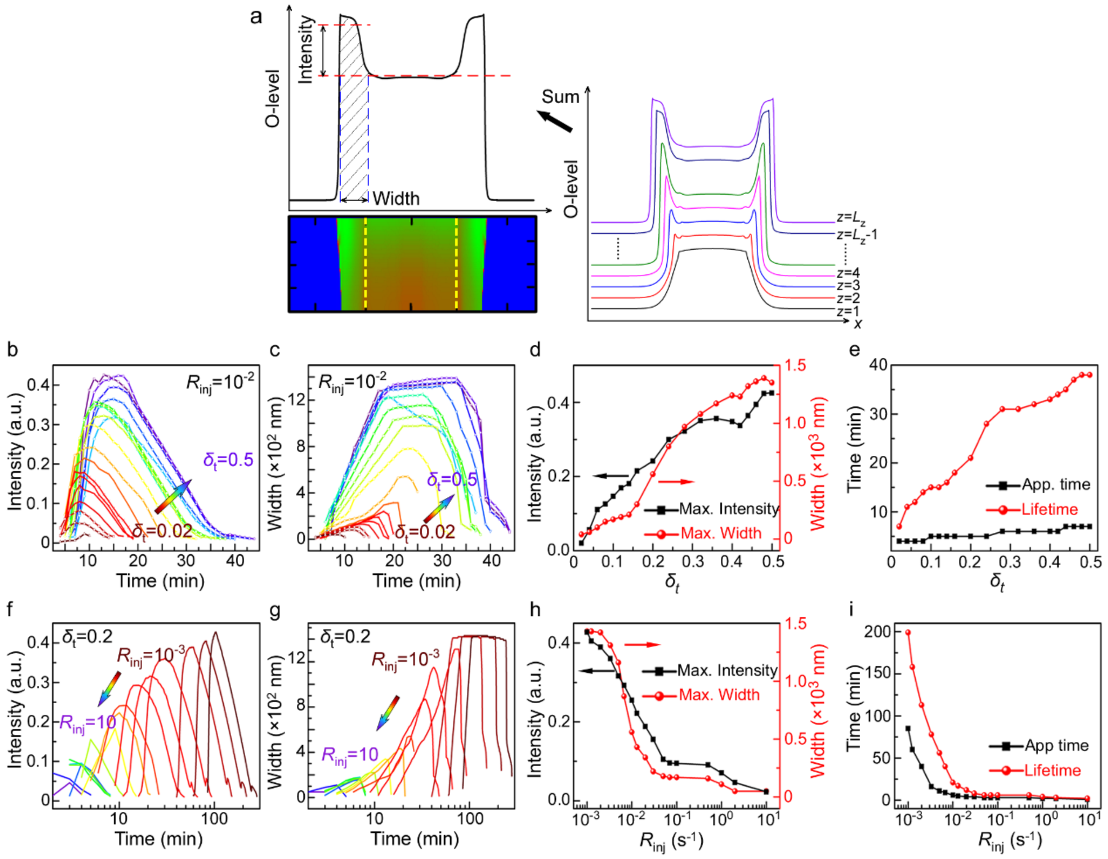

Supplementary Figure 19 | Sensitivity of simulation results to model parameters. **a**, Sketch of the O-level profiles along the *x* direction at various depths and their sum, which are used to estimate the properties of the HOR. The simulation step dependent **b**, intensity and **c**, width of the HOR for various δ_t values. The δ_t dependent **d**, maximum intensity (left axis) and width (right axis) of the HOR and **e**, App. time (left axis) and lifetime (right axis) of the HOR. The value of R_{inj} for **b–e** is 10^{-2} s^{-1}. The simulation step dependent **f**, intensity and **g**, width of HOR for various R_{inj}. The R_{inj} dependent **h**, maximum intensity (left axis) and width (right axis) of the HOR and **i**, App. time (left axis) and lifetime (right axis) of the HOR. The δ_t value for **f–i** is 0.2.

Supplementary Note 20. *Ex*-situ gating experiments and device preparation

For the *ex-situ* experiments of SQUID, XRD, and XAS, large area thin films of 5×2.5 mm^2 were used after gating for 30 mins in a Lake Shore probe stage with a background vacuum pressure of better than 10^{-6} mbar (Samples TF-A and TF-B). As shown in Supplementary Figure 20a, a small area of the thin film sample (total area of 6~7 mm×2.5 mm) with the contacts is not covered by the IL. After gating, this small ungated area is removed by wire-cutting so that the gated area of the samples for the *ex-situ* measurements is set to be 5×2.5 mm^2. Samples ST-A and ST-B for STEM (scanning TEM) measurements were also *ex-situ* gated in the same way as TF-A and TF-B, respectively.

Field effect transistor devices for transport measurements are in the form of Hall-bars with lateral gate electrodes located nearby the transistor channel. These devices were prepared by photo-lithography and wet etching (HCl and KI solution), as shown in Supplementary Figure 20b. The channel is 1000 µm long and 100 µm wide. Electrical contacts to the edge of the channel were made from Au (60 nm)/Cr (10 nm) that were deposited by thermal evaporation. The distance between the voltage contacts along the channel is 400 µm. The device preparation process is schematically shown in Supplementary Figure 20c. All the samples used in this study are from the same wafer of 40 nm (001) $SrCoO_{2.5}/SrTiO_3$.

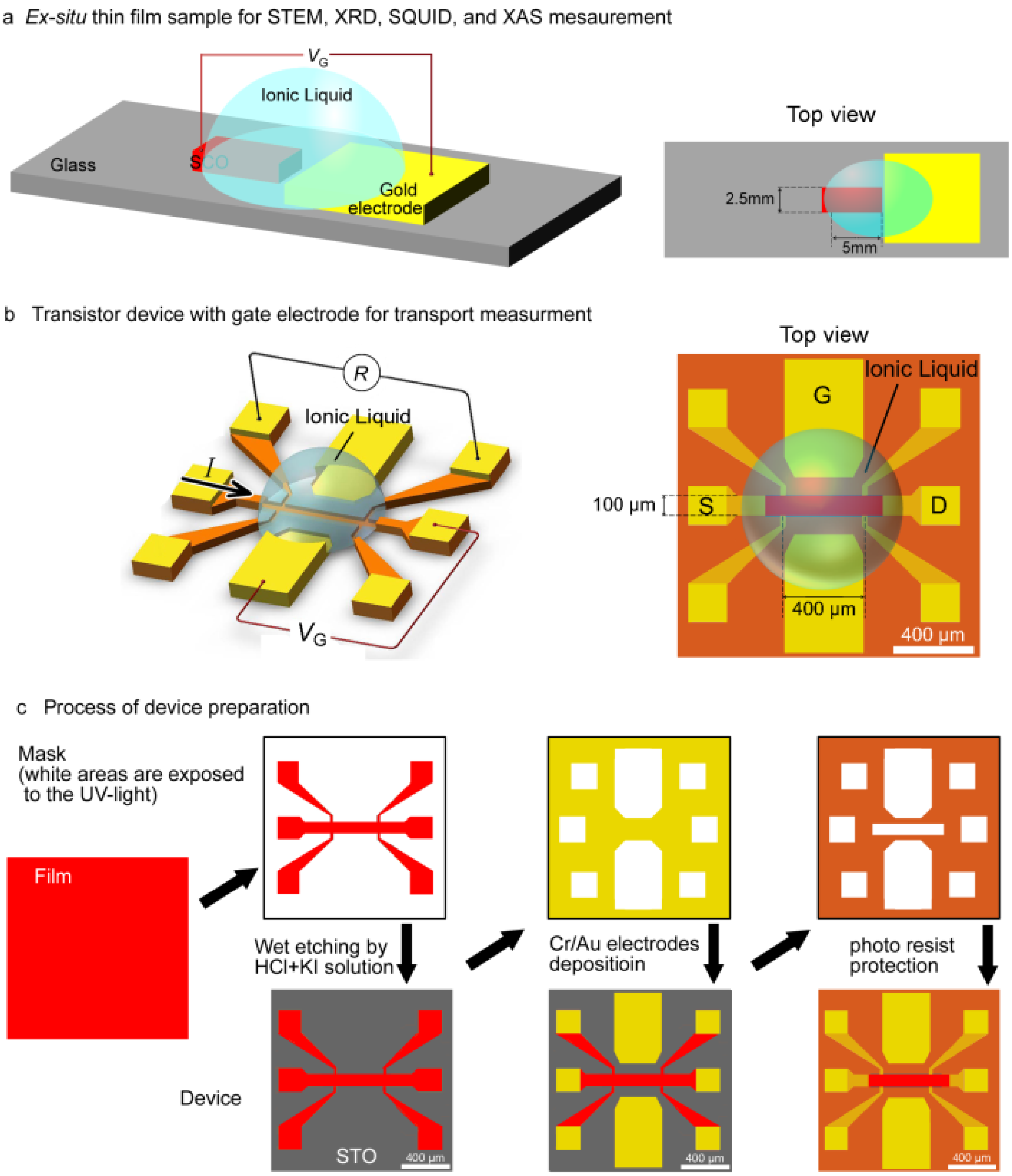

Supplementary Figure 20 | *Ex*-situ gating experiment and device preparation.

Schematic diagram of gating experiments for **a**, *ex-situ* gated thin film and **b**, IL gated transistor devices. **c**, Method of fabrication of the transistor.

Supplementary References

1. Jeen, H. *et al.* Reversible redox reactions in an epitaxially stabilized $SrCoO_x$ oxygen sponge. *Nat. Mater.* **12,** 1057–1063 (2013).

2. Yao, L., Majumdar, S., Akaslompolo, L., Inkinen, S., Qin, Q. H. & van Dijken, S. Electron-beam-induced perovskite-brownmillerite-perovskite structural phase transitions in epitaxial $La_{2/3}Sr_{1/3}MnO_3$ films. *Adv. Mater.* **26,** 2789–2793 (2014).

3. Moodenbaugh, A. R. *et al.* Hole-state density of $La_{1-x}Sr_xCoO_{3-\delta}$ ($0 \leqslant x \leqslant 0.5$) across the insulator/metal phase boundary. *Phys. Rev. B* **61,** 5666–5671 (2000).

4. Karvonen, L., Valkeapää, M., Liu, R.-S., Chen, J.-M., Yamauchi, H. & Karppinen, M. O-*K* and Co-*L* XANES study on oxygen intercalation in provskite $SrCoO_{3-\delta}$. *Chem. Mater.* **22,** 70–76 (2010).

5. Cui, B. *et al.* Insight into the antiferromagnetic structure manipulated by electronic reconstruction. *Phys. Rev. B* **94,** 134403 (2016).

6. Heggen, M., Luysberg, M. & Tillmann, K. FEI Titan 80-300 STEM. *Journal of large-scale research facilities* **2,** A42 (2016).

7. Jin, L., Jia, C.-L., Lindfors-Vrejoiu, I., Zhong, X., Du, H. & Dunin-Borkowski, R. E. Direct demonstration of a magnetic dead layer resulting from A-site cation inhomogeneity in a (La,Sr)MnO_3 epitaxial film system. *Adv. Mater. Interfaces* **3,** 1600414 (2016).

8. Wang, Z. Q., Zhong, X. Y., Yu, R., Cheng, Z. Y. & Zhu, J. Quantitative experimental determination of site-specific magnetic structures by transmitted electrons. *Nat. Commun.* **4,** 1395 (2013).

9. Jeong, J., Aetukuri, N., Graf, T., Schladt, T. D., Samant, M. G. & Parkin, S. S. P. Suppression of metal-insulator transition in VO_2 by electric field-induced oxygen vacancy formation. *Science* **339,** 1402–1405 (2013).

10. Cui, B. *et al.* Manipulation of electric field effect by orbital switch. *Adv. Funct. Mater.* **26,** 753–759 (2016).

11. Jeong, J., Aetukuri, N. B., Passarello, D., Conradson, S. D., Samant, M. G. & Parkin, S. S. Giant reversible, facet-dependent, structural changes in a correlated-electron insulator induced by ionic liquid gating. *Proc. Natl. Acad. Sci. USA* **112,** 1013–1018 (2015).

12. Chroneos, A., Yildiz, B., Tarancón, A., Parfitt, D. & Kilner, J. A. Oxygen diffusion in solid oxide fuel cell cathode and electrolyte materials: mechanistic insights from atomistic simulations. *Energy Environ. Sci.* **4,** 2774–2789 (2011).

13. Adler, S. B. Factors governing oxygen reduction in solid oxide fuel cell cathodes. *Chem. Rev.* **104,** 4791–4844 (2004).

14. Kim, Y. M. *et al.* Probing oxygen vacancy concentration and homogeneity in solid-oxide fuel-cell cathode materials on the subunit-cell level. *Nat. Mater.* **11,** 888–894 (2012).

15. Xie, C. K., Nie, Y. F., Wells, B. O., Budnick, J. I., Hines, W. A. & Dabrowski, B. Magnetic phase separation in $SrCoO_x$ ($2.5 \leqslant x \leqslant 3$). *Appl. Phys. Lett.* **99,** 052503 (2011).

16. Close, T., Tulsyan, G., Diaz, C. A., Weinstein, S. J. & Richter, C. Reversible oxygen scavenging at room temperature using electrochemically reduced titanium

oxide nanotubes. *Nat. Nanotechnol.* **10,** 418–422 (2015).

17. Nian, Y. B., Strozier, J., Wu, N. J., Chen, X. & Ignatiev, A. Evidence for an oxygen diffusion model for the electric pulse induced resistance change effect in transition-metal oxides. *Phys. Rev. Lett.* **98,** 146403 (2007).

18. Mefford, J. T. *et al.* Water electrolysis on $La_{1-x}Sr_xCoO_{3-\delta}$ perovskite electrocatalysts. *Nat. Commun.* **7,** 11053 (2016).

19. Zhang, Z. & Yates, J. T., Jr. Band bending in semiconductors: chemical and physical consequences at surfaces and interfaces. *Chem. Rev.* **112,** 5520–5551 (2012).

20. Lu, W. *et al.* Multi-nonvolatile state resistive switching arising from ferroelectricity and oxygen vacancy migration. *Adv. Mater.* **29,** 1606165 (2017).
